# Supplementary material for: Isolation of a New Polysaccharide from Dandelion Leaves and Evaluation of Its Antioxidant, Antibacterial, and Anticancer Activities
Source: Molecules. 2022 Nov 7;27(21):7641. doi: 10.3390/molecules27217641 (PMC9658512; doi:10.3390/molecules27217641)
Supplement: Supplementary file 1 [file molecules-27-07641-s001.zip › molecules-1978805-supplementary.pdf]

1. Supplementary methods:

Supplementary method S1:

Monosaccharide composition analysis

The DLP-3 (10 mg) was dissolved in 5 mL of trifluoroacetic acid (TFA, 2 M) and hydrolyzed at 110 °C for 8 h. Methanol was added into the hydrolysate and evaporated to dryness with rotary evaporator to remove TFA and repeated three times. Then, the mixture was dissolved in distilled water. Next, 100 μL hydrolysate was taken and mixed with 100 μL of NaOH solution (0.6 M), and added 100 μL 1-phenyl-3-methyl-5-pyrazolone (PMP) - methanol solution (0.5 M) and reacted at 70 °C for 100 min. Then the mixture was neutralized with HCl solution (0.3 M, 200μL) before dissolving with distilled water (1.4 mL). Finally, the mixture was extracted with chloroform and analyzed by HPLC (Agilent1100, USA).

The sample was separated by Eclipse Plus C18 column (4.6 × 250 mm, 5 μm) and column temperature was kept at 30 °C. The mobile phase was composed of 0.1 M phosphate buffer (pH 6.7) and acetonitrile (87:13, *v/v*) with a flow rate of 1.0 mL/min. The UV detection wavelength was set at 245 nm.

Supplementary method S2:

Methylation analysis

Briefly, DLP-3 (5mg) was dissolved in 5 mL of anhydrous dimethyl sulfoxide (DMSO) before NaOH (100 mg) was added. After the mixture was stirred for 4 h, methyl iodide (1.5 mL) was added and the mixture was stored for 2 h at 25 °C in the dark, and distilled water (4 mL) was added. The products were extracted three times with chloroform (3 mL), repeating until the methylation was complete.

The methylated DLP-3 was hydrolyzed with 10 mL trifluoroacetic acid (TFA) (2 mol/L) at 100 °C for 8 h. Then the hydrolysates were dissolved into 4 mL of 1% (*w/w*) NaOH and 10 mg $NaBH_4$ was added to reduce hemiacetal bond and 100 μL of glacial acetic acid was added after the reduction. The sample was dried under reduced pressure, followed by acetylation with acetic anhydride (1.5 mL) and pyridine (1.5 mL) at 100 °C for 2 h.The final reaction products were extracted with 4 mL of chloroform and analyzed by GC-MS (Agilent 6890-5973 N, Agilent, Santa Clara, CA, USA) with an Agilent HP-5MS capillary column. The temperature program was increased from 140 °C to 200 °C at 10 °C/min, hold for 5 min, then increased to 240 °C at 8 °C/min. The injection temperature was 250 °C, the split ratio was 50:1, and the injection volume was 5 μL.

Supplementary method S3:

Superoxide radical scavenging assay

Briefly, 2 mL of Tris−HCl buffer (50 mM, pH 8.2) was added to 1 mL of various concentrations (0.2, 0.4, 0.6, 0.8 and 1 mg/mL) of DLP-3, then 0.2 ml of pyrogallol (6 mM) was added. The mixture was shaken vigorously and reacted at 25 °C for 5 min. Finally, 0.5 ml of HCl (0.1 M) was added to terminate the reaction. Absorbance was determined at 320 nm. Vc was used as the positive control. The superoxide radical scavenging effect of DLP-3 was calculated as follows :

$$\text{Superoxide radical scavenging effect}\ (\%) = \left(1 - \frac{A_2 - A_3}{A_0 - A_1}\right) \times 100\% \quad (1)$$

Where A_0 represents the absorption of the negative control (superoxide system with distilled water), A_1 represents the absorption of the system background (Tris−HCl buffer with distilled water), A_2 represents the absorption of the sample (superoxide system with samples), and A_3 represents the absorption of the sample background (Tris−HCl buffer with samples).

Supplementary method S4:

ABTS radical scavenging assay

In short, 5mL of ABTS solution (7mM) was mixed with 5mL of potassium persulfate (2.45 mM), and the solution incubated in dark for 12 h under room temperature. The ABTS free radical solution was diluted with phosphate buffer (pH 6.6), with adjusted absorbance to 0.70 ± 0.02 at 734 nm. Then 0.1 mL of various concentrations (0.2, 0.4, 0.6, 0.8 and 1 mg/mL) of DLP-3 was added into 3.9 mL of ABTS free radical solution. The mixture was well shaken, and the absorbance was measured (734 nm) within 6 min. Vc was used as the positive control. The ABTS scavenging effect was estimated as follows:

$$\text{ABTS scavenging activity } (\%) = \left(1 - \frac{A_1 - A_2}{A_0}\right) \times 100\% \quad (2)$$

Where A_0 is the absorbance of the control solution (without sample); A_1 is the absorbance of the sample solution (sample and ABTS); A_2 is the absorbance of the background solution (without ABTS).

Supplementary method S5:

Hydroxyl radical scavenging assay

Briefly, 1mL of $FeSO_4$ solution (9 mM), 1mL of salicylic acid-ethanol solution (9 mM), and 1 mL of H_2O_2 solution (1 mg/mL) was added to 1 mL of various concentrations (0.2, 0.4, 0.6, 0.8 and 1 mg/mL) of DLP-3. The mixture was shaken and reacted at 37 °C for 30 min. Absorbance was determined at 510 nm. Vc was used as the positive control. The scavenging activity was estimated as follows:

$$\text{Hydroxyl radical scavenging effect } (\%) = \left(1 - \frac{A_1 - A_2}{A_0}\right) \times 100\% \quad (3)$$

Where A_0 is the absorbance of the control (without sample); A_1 is the absorbance of the sample solution; A_2 is the absorbance of the sample background (without H_2O_2).

Supplementary method S6:

Cell proliferation assay and colony formation assay

At first, the HepG2 cells at the density of 1×10^4 (cells/well) were seeded to each well of 96-well plates with 100 μL culture medium in each well and maintained in 5% CO_2 humidified atmosphere at 37 °C for 24 h. After that, the cells were treated with DLP-3 at different concentrations (0, 50, 100, 200, 400 and 800 μg/mL) or DMSO (vehicle) for 0, 24, 48, 72, and 96 h, respectively. Then, the medium from each well was removed and 100 μL of MTT solution (5 mg/mL in PBS) was added to each plate and incubated for 4 h. Finally, the medium was carefully replaced with 150 μL DMSO per well. Absorbance was determined at 492 nm by a microplate reader (MK-3, Thermo, USA) and untreated cells was used as control. The inhibitory rate of tumor cells (IR_{TC}) was calculated as a percentage as follows:

$$IR_{TC} (\%) = \left(1 - \frac{\text{Asample}}{\text{Acontrol}}\right) \times 100\ \% \quad (4)$$

Asample and Acontrol are the absorbance of treated cells and untreated cells.

HepG2 cells were seeded in 6-well plates (800 cells/well) and cultured for 14 days until visible colonies were formed. The colonies were fixed and stained with crystal violet. After staining, the plates were washed and air-dried, and the colony numbers were counted. Each experiment was repeated in triplicate.

2. Supplementary Figures:

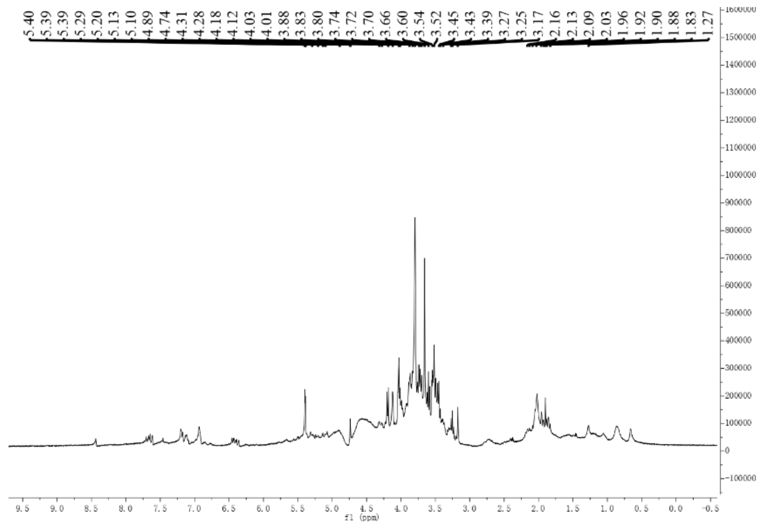

Figure S1. ^{1}H NMR spectrum of DLP-3.

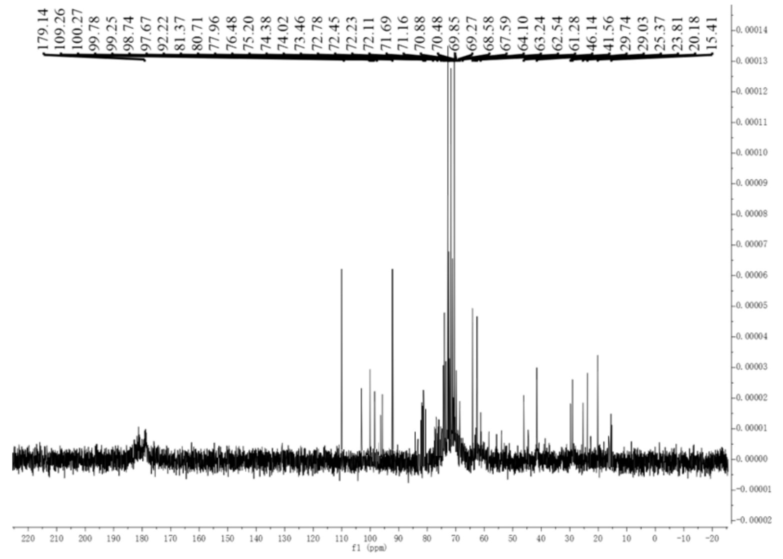

Figure S2. 13C NMR spectrum of DLP-3.
